# Increased Phosphorylation of Intracellular Signaling Molecules Indicates Continuous Activation of Human Autoreactive B‐Cells

**DOI:** 10.1002/eji.202451361

**Published:** 2025-01-16

**Authors:** Sanne Kroos, Nienke J. Blomberg, Joanneke C. Kwekkeboom, Rudi W. Hendriks, Odilia B. J. Corneth, René E. M. Toes, Hans U. Scherer

**Affiliations:** ^1^ Department of Rheumatology Leiden University Medical Center Leiden The Netherlands; ^2^ Department of Pulmonary Medicine Erasmus MC University Medical Center Rotterdam The Netherlands

**Keywords:** autoimmunity, B cells, cellular immunology, kinases, rheumatology

## Abstract

Many human autoimmune diseases (AIDs) are hallmarked by the presence and persistence of autoreactive B‐cells. While autoreactive B‐cells may frequently encounter antigens, the signals required to balance and maintain their activation and survival are mostly unknown. Understanding such signals may be important for strategies aimed at eliminating human B‐cell autoreactivity. Here, we assessed intracellular signaling pathways in B cells targeting citrullinated protein antigens isolated from patients with rheumatoid arthritis (RA), a common and well‐characterized AID. Peripheral blood mononuclear cells of 15 RA patients positive for anti‐citrullinated protein antibodies (ACPA) were analyzed directly ex vivo using spectral flow cytometry and B‐cell differentiation markers, citrullinated antigen‐biotin‐streptavidin tetramers, and intracellular (phosphoflow) markers. Tetanus toxoid (TT)‐specific B cells served as antigen‐specific comparators. In absence of any in vitro BCR stimulation, ACPA‐expressing memory B cells (MBCs) displayed enhanced expression of Ki‐67 and increased SYK‐, BTK‐, AKT‐, and S6‐phosphorylation compared with TT‐specific MBCs. We demonstrate the simultaneous detection of B cell antigen‐specificity and intracellular protein phosphorylation on the single‐cell level. The data reveal that autoreactive B‐cells in RA, in contrast to B cells against recall antigens, display enhanced phosphorylation of signaling molecules that point toward continuous, presumably antigen‐mediated activation of the autoreactive B‐cell compartment.

## Graphical abstract

Directly ex‐vivo and after fixation, autoreactive B cells were detected using fluorescent streptavidin molecules conjugated to citrullinated or arginine peptides. Anti‐tetanus toxoid B cells served as controls and were detected using fluorescently labeled tetanus toxin. Antigen‐specific phosphoflow revealed increased levels of pSYK, pBTK, pAKT, and pS6 in autoreactive B cells.

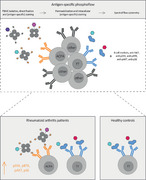

**Abbreviations**
ACPA B cells: ACPA‐expressing B cells
ACPA: anti‐citrullinated protein antibodies
AIDs: autoimmune diseases
AKT: serine/threonine‐specific protein kinase
BAFFR: B‐cell activating factor receptor
BCR: B‐cell receptor
BTK: Bruton's tyrosine kinase
ESR: erythrocyte sedimentation rate
MBCs: memory B cells
MFI: median fluorescent intensity
PB: plasmablasts
PBMCs: peripheral blood mononuclear cells
RA: rheumatoid arthritis
SYK: spleen tyrosine kinase
TLR: toll‐like receptors
TT: tetanus toxoid

## 1 Introduction

Most human autoimmune diseases (AIDs) are chronic diseases without a prospect for cure. Current therapeutics effectively suppress inflammation but fail to target the underlying cause of disease. Rheumatoid arthritis (RA) is characterized by chronic inflammation and synovial joint destruction. RA is responsive to therapeutic B‐cell depletion, indicating a pivotal role for B cells in disease pathogenesis [1]. Anti‐citrullinated protein antibodies (ACPA) are a hallmark of RA, but the pathogenicity of these autoantibodies is under debate. Nevertheless, evidence indicates that ACPA‐expressing B‐cells (ACPA B‐cells) may be important drivers of disease. These cells display an activated and proliferative phenotype throughout the course of RA [2]. The continuous activation of autoreactive B‐cells is thought to reflect ongoing immunological activity in AID patients and could explain frequent disease flares when treatment is stopped. Possible causes of autoreactive B‐cell activation include, but are not limited to, cytokines present in proinflammatory environments, innate triggers via toll‐like receptors (TLRs), or (auto)antigen recognition. Here, we aimed to study signaling pathways involved in the chronic activation of autoreactive B cells, using ACPA B‐cells as a well‐defined prototype response.

B‐cell activation is mediated by multiple receptors and a complex interplay between inhibitory and stimulatory signals [3]. B‐cell receptor (BCR) crosslinking activates protein tyrosine kinases such as spleen tyrosine kinase (SYK), Bruton's tyrosine kinase (BTK), the serine/threonine‐specific protein kinase AKT (AKT), and ribosomal protein S6. BTK, and to some extent SYK, are also involved in signaling pathways downstream of TLRs and chemokine receptors [4, 5]. AKT‐ and subsequent S6‐phosphorylation can occur through BCR‐, TLR‐, and B‐cell activating factor receptor (BAFFR)‐engagement [6, 7]. Hence, the level of (differential) phosphorylation of signaling kinases in autoreactive B‐cells may identify pathways operational during chronic activation. Insights may be clinically relevant as various kinase inhibitors are evaluated as therapeutics for several AIDs, with varying success [8].

Phosphoflow combines detecting phosphorylated proteins with flow cytometry and allows investigating intracellular protein phosphorylation at the single‐cell level in rare B‐cell populations [9]. Studies so far investigated differences in protein phosphorylation upon in vitro B‐cell stimulation and focused on the total B‐cell compartment and its subsets. Investigating rare, antigen‐specific B‐cells remained challenging as their identification requires BCR ligation. Here, we combined phosphoflow with a sensitive, antigen‐specific B‐cell staining approach upon direct ex vivo isolation of peripheral blood mononuclear cells (PBMCs) to identify and evaluate signaling cascades in autoreactive and nonautoreactive B cells in individual patients. We observed enhanced SYK, BTK, AKT, and S6 phosphorylation in ACPA memory B‐cells (MBCs) compared with TT‐specific MBCs. This finding indicates the recent triggering of the ACPA BCR and, likely, signaling via additional receptors.

## 2 Results and discussion

### 2.1 Measuring Phosphorylation Levels in Antigen‐Specific B Cells Without Triggering the BCR

**FIGURE 1:**
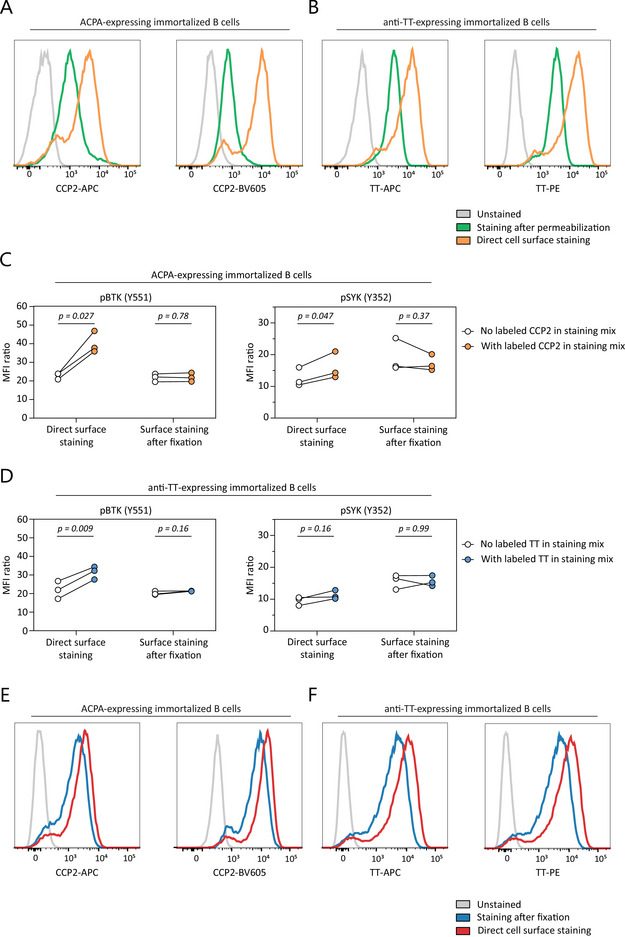
Protocol optimization on immortalized B cell lines expressing membrane‐bound ACPA (A, C, E) or anti‐TT (B, D, F) BCRs. (A, B) Antigen‐specific signal resolution upon staining after cell permeabilization or directly after cell surface staining. Data are representative of three independent experiments. (C, D) pBTK and pSYK signals upon antigen‐specific staining while keeping cells on ice, with and without prior fixation. Each individual datapoint represents the mean of two technical replicates. Each linked datapoint represents one independent experiment: in total, three independent experiments were performed. (E, F) Antigen‐specific signal resolution when cells are stained after fixation or directly after surface staining. Data are representative of three independent experiments. All p‐values were calculated with a Wilcoxon signed rank test.

Pathogenic B‐cell memory and its ability to generate plasmablasts (PBs) and plasma cells upon activation forms an integral part of many human AIDs. To analyze signaling molecule phosphorylation in autoreactive B‐cells at the single‐cell level, we developed a combined protocol for autoreactive B‐cell identification [10] and phosphorylation analysis of signaling molecules by phosphoflow [11]. To this end, we first used immortalized B cells expressing membrane‐bound ACPA or anti‐TT BCRs and stained these cells with fluorescently labeled antigens. Antigen‐specific staining after cell permeabilization led to a decrease in signal indicating a substantial loss of sensitivity due to the procedure (Figure 1A,B). To overcome this effect, all extracellular antigens (including BCRs) were stained prior to permeabilization. Labeled antigens binding the BCR, however, induced a clear increase in pBTK and pSYK expression despite performing the experimental procedures strictly on ice (Figure 1C,D). Therefore, a separate fixation step was added prior to extracellular staining to avoid the induction of BCR signaling while maintaining adequate sensitivity for autoreactive B‐cells. This yielded a clear antigen‐specific signal (Figure 1E,F) without increasing pSYK or pBTK expression (Figure 1C,D).

### 2.2 Frequencies and Phosphorylation Levels Throughout B‐Cell Subsets

**FIGURE 2:**
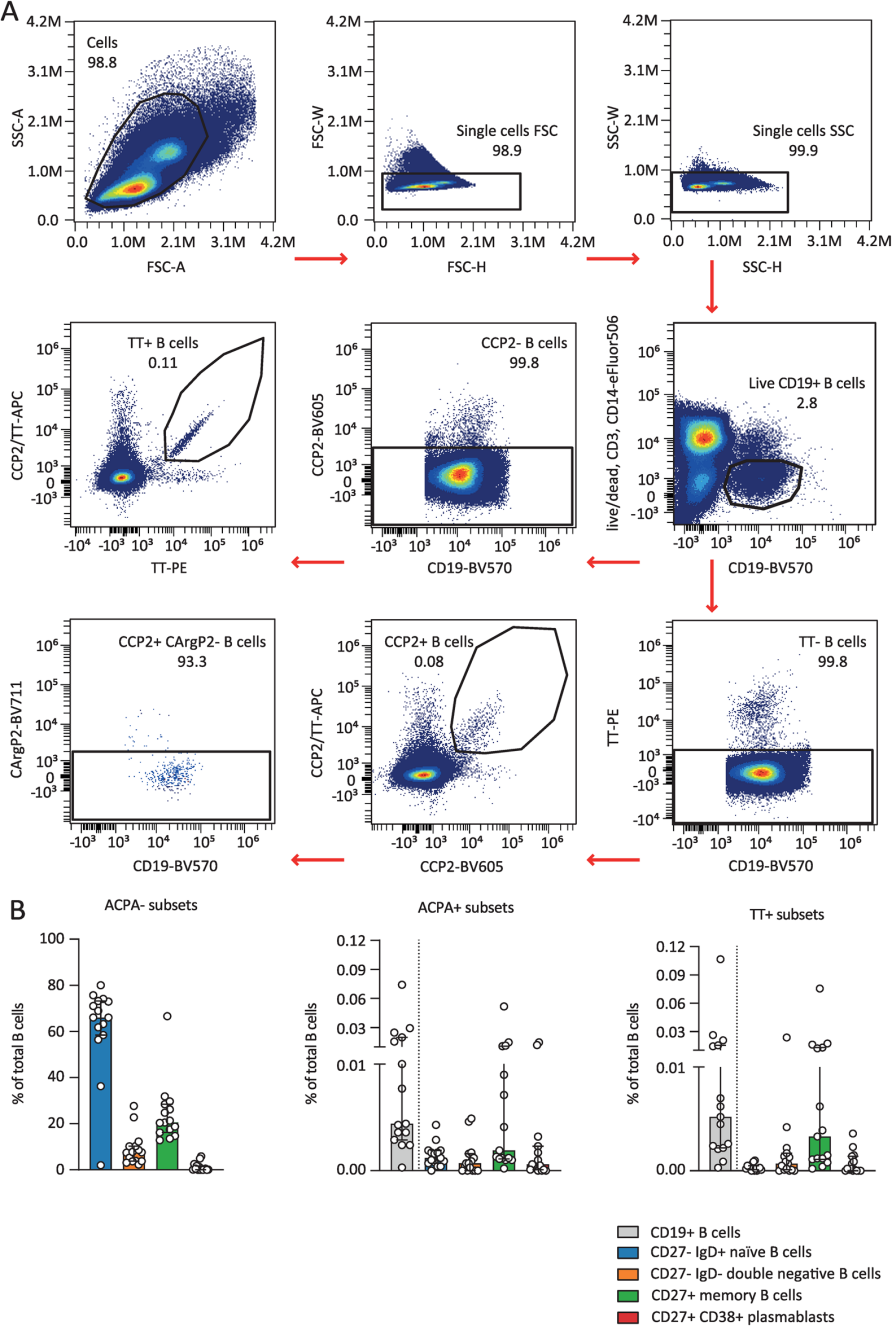
Antigen‐specific B cell frequencies. (A) Gating strategy for antigen‐specific B‐cell identification of one representative RA patient sample. Selected ACPA B cells were negative for the CArgP2 control antigen. (B) Bar graphs depicting subset frequencies. Each dot represents one patient. Figures show pooled data from 15 patients analyzed in separate experiments. Medians and interquartile ranges are indicated. Dashed line emphasizes that CD19+ B cells (gray) are the total of the other populations.

To assess the phosphorylation state of primary antigen‐specific B cells, we next applied the protocol to PBMCs from 15 ACPA‐positive RA patients and five healthy donors. Patient and healthy donor characteristics are provided in Table S1. Fluorescently‐labeled antigens were added to the intracellular staining mix to also detect antigen‐specific antibody‐secreting cells [12]. Cell populations were gated as visualized (Figure S1A) to divide B cells into four distinct subsets. SYK, BTK, AKT, and S6 phosphorylation levels were assessed on ACPA‐negative B cells for reference (Figures S1B and S2D). Additional gating identified ACPA‐expressing and TT‐specific B cells (Figure 2A). Frequencies in these antigen‐specific subsets were consistent with previous findings (Figure 2B and Figure S2C) [2, 10].

### 2.3 Enhanced Phosphorylation Levels in ACPA‐Expressing MBCs

**FIGURE 3:**
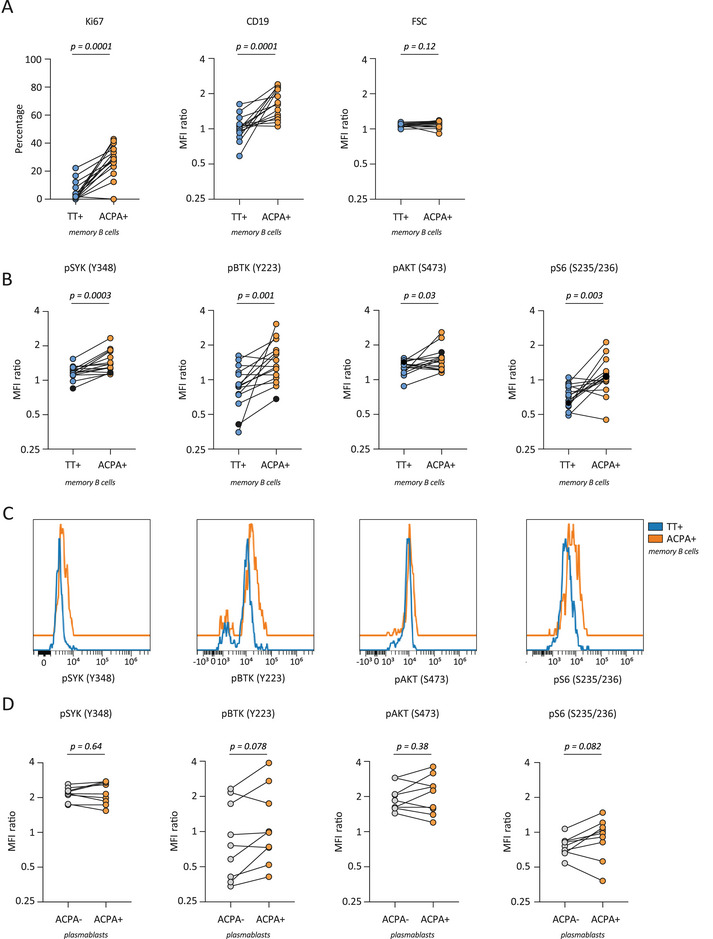
Phosphorylated signaling kinases in ACPA MBCs compared with TT‐specific MBCs. (A) Levels of Ki67 and MFI ratios of CD19 and FSC in ACPA MBCs. (B) Levels of pSYK, pBTK, pAKT and pS6 in ACPA MBCs. (C) Histograms depicting MFIs from a representative sample (black dots in B). (D) Levels of signaling kinases in ACPA PBs compared with ACPA‐negative PBs. Only patients with detected ACPA PBs were analyzed (*n* = 9). Each dot represents one patient. Figures show pooled data from 15 patients analyzed in separate experiments. All *p*‐values were calculated with Wilcoxon signed rank test.

ACPA MBCs are highly active and proliferative in established disease as evidenced by enhanced expression of Ki67 and CD19 [2] (Figure 3A), expression of co‐stimulatory ligands, secretion of proinflammatory cytokines [2], generation of CXCR3+ PBs [12], and their presence as differentiated PBs in the synovial compartment. Interestingly, this activated state was reflected by enhanced intracellular kinase phosphorylation in most donors. In fact, compared with TT‐specific MBCs representing a resting MBC response, elevated phosphoflow signals were observed for all four signaling molecules analyzed, SYK, BTK, AKT, and S6 (Figure 3B,C). Of these, only pSYK but not pBTK, pAKT, or pS6 correlated with Ki67 expression (Figures S3C–F). Moreover, when compared with the total pool of ACPA‐negative MBCs, all signaling molecules showed enhanced expression by ACPA+ MBCs, except for pBTK (Figure S3B). Importantly, in contrast to previous studies that applied phosphoflow cytometry to larger B cell populations, we observed these differences in primary B cells isolated directly ex vivo without in vitro stimulation. Thus, these data closely reflect the state of cellular activation of the autoreactive MBC compartment in vivo and the signaling pathways operational at the moment of isolation. In PBs, the degree of phosphorylation was similar between ACPA‐negative and ACPA‐expressing cells (Figure 3D). To assess whether the inflammatory environment in RA patients could impact the protein phosphorylation observed, we additionally assessed phosphorylated proteins of TT‐specific MBCs and total MBCs in healthy donors (Figure S2A,B). The levels of phosphorylated protein expression in healthy donors were highly similar to those observed in patients with RA, suggesting that the influence of the inflammatory environment is likely minimal.

These data together with previous phenotypic evaluations are consistent with the notion that ACPA MBCs resemble recent germinal center emigrants. The kinases investigated are known to be involved in BCR downstream signaling, although the extensive interplay in signaling pathways of various receptors does not allow the increased activation to be pinpointed to one single cause. Fc receptors, integrins, and BAFFR, for example, all employ SYK and BTK in their signaling pathways [7, 13, 14]. ACPA adds a layer of complexity to this system, as ACPA variable domains are heavily glycosylated [15]. These variable domain glycans enhance BCR signaling upon antigen binding [16]. So far, it is unknown whether variable domain glycans can also cause antigen‐independent, tonic BCR signaling.

Our work is of relevance for the design and application of future therapeutic interventions. Increased CD19 and pBTK levels in ACPA MBCs indicate the CD19/BTK axis as a possible target for therapy. CD19 lowers the threshold of BCR signaling by amplifying and prolonging BTK activation [17]. Enhanced BCR signaling caused by CD19 overexpression promotes loss of tolerance toward self‐antigens in mice, as well as autoantibody production by plasmablasts and plasma cells [18, 19]. In preclinical models, BTK inhibition diminishes inflammatory responses and histological damage [20]. Moreover, BTK protein and pBTK are enhanced in the total B‐cell population of ACPA‐positive compared with ACPA‐negative RA patients [21]. Nevertheless, clinical trials investigating the efficacy of BTK‐inhibitors in RA patients reported differential clinical responses [20]. Also, a phase II clinical trial evaluating fostamatinib, a SYK inhibitor, resulted in significantly improved response rates when administered with methotrexate [22]. This effect, however, was less pronounced in the phase III trial [23]. To what extent antigen‐specific SYK or BTK inhibition could be beneficial remains to be determined. A similar targeted therapy approach may also be applied to rapamycin, an mTOR inhibitor that demonstrated alleviation of RA symptoms [24]. Its broad reactivity poses a challenge as it compromises the overall immune defense, arguing in favor of future selective, antigen‐specific B‐cell targeting strategies [24].

## 3 Data Limitations and Perspectives

Our study has limitations. Sample numbers and the number of signaling molecules assessed are small and represent a first exploration rather than a detailed analysis of individual pathways. Several signaling molecules of interest require permeabilization buffers that are not compatible with all fluorochromes and thus require additional panel design [11]. In addition, causal relationships or the identification of single receptor stimulation were not addressed. Furthermore, experiments were performed with freshly isolated PBMCs and potential effects from cryopreservation have not been investigated. Finally, more stringent patient selection will be necessary to yield insights into possible treatment effects on the immunological parameters assessed.

## 4 Concluding Remarks

In summary, this manuscript describes a novel approach to investigate signaling kinases in rare, antigen‐specific human B cells. Our data reveal that circulating ACPA MBCs, in contrast to TT‐specific MBCs, display increased kinase phosphorylation if isolated directly ex vivo, pointing to active BCR‐mediated signaling and additional modulators. This possibly indicates recent antigen encounter and/or involvement in the defective clonal deletion of these autoreactive B cells. Together, these analyses and the broad applicability of the approach to antigen‐specific cellular immune responses enhance our understanding of disease mechanisms operational in chronic AIDs.

## 5 Materials and Methods

### 5.1 Antigen Labeling

Biotinylated cyclic citrullinated peptide (CCP)2 was conjugated to brilliant violet 605 (BV605)‐ or allophycocyanin (APC)‐labeled streptavidins [10]. The arginine control variant CArgP2 was conjugated to brilliant violet 711 (BV711)‐labeled streptavidin. TT protein (Statens Serum Institute) was labeled with APC and R‐Phycoerythrin (PE) using the AnaTag labeling kit (AnaSpec). Titrations of the labeled tetramers and proteins were performed on immortalized B cells expressing either an ACPA BCR or anti‐TT BCR to determine optimal concentrations for antigen‐specific B‐cell identification.

### 5.2 Immortalized B Cells

Immortalized B cells expressing either membrane‐bound ACPA‐IgG or anti‐TT‐IgG were generated by transduction of primary human memory B cells with Bcl‐6 and Bcl‐xL, as previously described [25]. Immortalized B cells were cultured in B‐cell‐culture medium (IMDM [Gibco], 8% FCS, 100 U/mL penicillin/streptomycin, 2 mM GlutaMAX [Gibco]) with irradiated mouse L cell fibroblasts expressing CD40L (CD40L‐L‐cells) and 25 ng/mL mouse IL‐21‐Fc. Per milliliter of culture medium, 5 × 10^5^ immortalized B cells were cultured together with 5 × 10^4^ CD40L‐L‐cells and IL‐21‐Fc.

Immortalized B cells were harvested and stained side by side following two protocols designed to assess the effects of antigen‐specific staining on the phosphorylation of proteins downstream of the BCR.

Cell fraction one was washed twice with PBS to cool the cells down and were kept carefully on ice thereafter during incubation for 30 min with an extracellular staining mix containing CCP2‐APC and CCP2‐BV605 or TT‐APC and TT‐PE. Cells were then washed twice with cold PBS/BSA 1%, resuspended in PBS/BSA, and incubated with an equal amount of fixation/permeabilization buffer (37°C, eBioscience #00‐5523‐00) for 10 min at 37°C as the cells were still viable during this permeabilization step.

Cell fraction two was washed twice with PBS, then directly resuspended in fixation buffer (BD Cytofix #554655, diluted 2x with PBS), and incubated for 5 min on ice. Thereafter, cells were washed twice with cold PBS/BSA and incubated for 30 min at room temperature (RT) in extracellular staining mix containing CCP2‐APC and CCP2‐BV605 or TT‐APC and TT‐PE. Cells were then washed twice with PBS/BSA at RT and resuspended in fixation/permeabilization buffer (eBioscience #00‐5523‐00) to incubate for 10 min at RT since the cells were already fixed and maintained at RT during previous steps.

Both cell fractions were treated identically for the following steps. Following fixation/permeabilization, samples were washed once with permeabilization washing buffer (eBioscience #00‐5523‐00) and incubated for 30 min at RT in intracellular staining mix containing pSYK‐PE‐Cy7 (17A/P‐ZAP70, BD) and pBTK‐PerCP‐eFluor710 (M4G3LN, ThermoFisher). Cells were then washed twice with permeabilization washing buffer, resuspended in 1% PFA buffer, and incubated for 15 min at RT. Finally, cells were washed and resuspended in PBS/BSA and stored at 4°C until measurement.

### 5.3 Patients and Healthy Donors

Peripheral blood was collected from 15 ACPA‐positive RA patients recruited from the outpatient clinic of the Department of Rheumatology at Leiden University Medical Center (LUMC). All patients met the 2010 American College of Rheumatology/European League Against Rheumatism (ACR/EULAR) criteria for RA at the time of diagnosis. The ethical review board of the LUMC gave permission to conduct the study (protocol P17.151) and all patients provided written informed consent. Included patients were treatment naïve or received methotrexate‐, hydroxychloroquine, and/or sulfasalazine treatment (Table S1). Peripheral blood from five healthy donors was obtained through the LUMC Voluntary Donor Service Biobank and written informed consent was obtained from all donors.

### 5.4 Antigen‐Specific Phosphoflow Staining (Optimized Protocol)

RA patient PBMCs were isolated by Ficoll‐Paque gradient centrifugation and immediately used for subsequent experimental steps. First, the PBMCs were incubated with Fixable Viability Dye eFluor506 (Invitrogen) for 30 min on ice in the dark. Thereafter, cells were washed twice with PBS/BSA 1% and incubated for 5 min in Cytofix fixation buffer (BD Cytofix #554655, 2x diluted in PBS) on ice. Cells were washed twice with PBS/BSA and kept overnight at 4°C in a B‐cell culture medium (IMDM, 8% FCS, 100 U/mL penicillin/streptomycin). The next day, cells were washed twice in PBS/BSA and resuspended in an extracellular staining mix (CD3‐eFluor506 (UCHT1, Invitrogen), CD14‐eFluor506 (61D3, Invitrogen; live/dead, CD3 and CD14 made up the dump channel), CD19‐BV570 (HIB19, BioLegend), CD27‐APC‐Fire810 (QA17A18, BioLegend), CD38‐BV785 (HIT2, BioLegend), IgD‐APC‐Fire750 (IA6‐2, BioLegend), CCP2‐APC, CCP2‐BV605, CArgP2‐BV711, TT‐APC, TT‐PE and incubated for 30 min at RT. Cells were subsequently washed twice with PBS/BSA, resuspended in fixation/permeabilization buffer (eBioscience #00‐5523‐00) and incubated at RT for 10 min. Cells were again washed once in permeabilization washing buffer (eBioscience #00‐5523‐00), resuspended in an intracellular staining mix containing Ki‐67‐PE‐Cy7 (20Raj1, ThermoFisher), pSYK‐AF488 (I120‐722, BD), pBTK‐BV421 (N35‐86, BD), pAKT‐PE‐CF94 (M89‐61, BD), pS6‐AF700 (D57.2.2E, CST), CCP2‐APC, CCP2‐BV605, CArgP2‐BV711, TT‐APC, TT‐PE. After 30 min incubation at RT, cells were washed twice with permeabilization washing buffer, resuspended in 1% PFA, and incubated for 15 min at RT. Finally, cells were washed and resuspended in PBS‐BSA and stored at 4°C until measurement on a Cytek 3L Aurora flow cytometer.

### 5.5 CCP2‐IgG ELISA

CCP2‐IgG levels in patient plasma were determined by ELISA. Biotinylated CCP2 or the native control variant CArgP2 were added to wells coated with streptavidin (Invitrogen) in a 384‐well plate (Corning). Plasma samples were tested in a 1:50, 1:400, or 1:3200 dilution, and reactivity was detected by HRP‐conjugated polyclonal rabbit anti‐human IgG visualized with ABTS + H_2_O_2_ (Sigma‐Aldrich). Absorbance was measured at 415 nm using SpectraMax i3x (Molecular Devices).

### 5.6 Data Analyses

Flow cytometry data were analyzed using OMIQ software. For experiments with immortalized cells, MFI ratios were determined by normalization to the MFI of an unstained sample. For patient samples, ratios were calculated by normalization to ACPA‐negative CD27‐IgD+ naïve B cells. Statistical analyses were performed using GraphPad Prism 9.3.1.

## Author Contributions

Sanne Kroos, Nienke J. Blomberg, Rudi W. Hendriks, Odilia B. J. Corneth, René E. M. Toes, and Hans U. Scherer: Conceptualization, design, and interpretation of the data. Sanne Kroos, Nienke J. Blomberg, and Joanneke C. Kwekkeboom: Acquisition and analysis of the data. Sanne Kroos, Nienke J. Blomberg, and Hans U. Scherer: Drafting the manuscript. All authors read and approved the final manuscript.

## Conflicts of Interest

The authors declare no conflicts of interest.

### Peer Review

The peer review history for this article is available at https://publons.com/publon/10.1002/eji.202451361

## Supporting information



Supplementary information

## Data Availability

The data supporting the findings of this study are available from the corresponding author upon reasonable request.
