# Supplementary material for: Increased Phosphorylation of Intracellular Signaling Molecules Indicates Continuous Activation of Human Autoreactive B‐Cells
Source: Eur J Immunol. 2025 Jan 16;55(1):e202451361. doi: 10.1002/eji.202451361 (PMC11739663; doi:10.1002/eji.202451361)
Supplement: Supplementary file 1 — Supplementary information [file EJI-55-e202451361-s001.docx]

**Supplementary table 1: Patient characteristics.**

| **Characteristic** | **RA patients (n = 15)** | **Healthy donors (n = 5)** |
| --- | --- | --- |
| Age, years* | 67 (62 – 70) | 60 (56 – 67) |
| Female, n (%) | 12 (80%) | 4 (80%) |
| CCP2-IgG (aU/mL)* | 1543 (244 – 2578) | n/a |
| DAS28(3v)* | 3,4 (2,2 – 3,8) | n/a |
| 28 swollen joint count* | 1 (0 – 2) | n/a |
| 28 tender joint count* | 1 (0 – 2) | n/a |
| Erythrocyte sedimentation rate (mm/hr)* | 33 (14 – 39) | n/a |
| Current treatment |  |  |
| Untreated, n | 2 | n/a |
| Methotrexate, n | 11 | n/a |
| Hydroxychloroquine, n | 2 | n/a |
| Sulfasalazine, n | 2 | n/a |
| Analyzed memory B cells* |  |  |
| ACPA | 21 (14 – 57) | n/a |
| TT | 24 (7 – 194) | 73 (39 – 205) |
| Analyzed ACPA plasmablasts* (n = 9 patients) | 14 (7 – 44) | n/a |
| *Values represent median (interquartile range). RA, rheumatoid arthritis. DAS, disease activity score. n/a, not applicable | | |


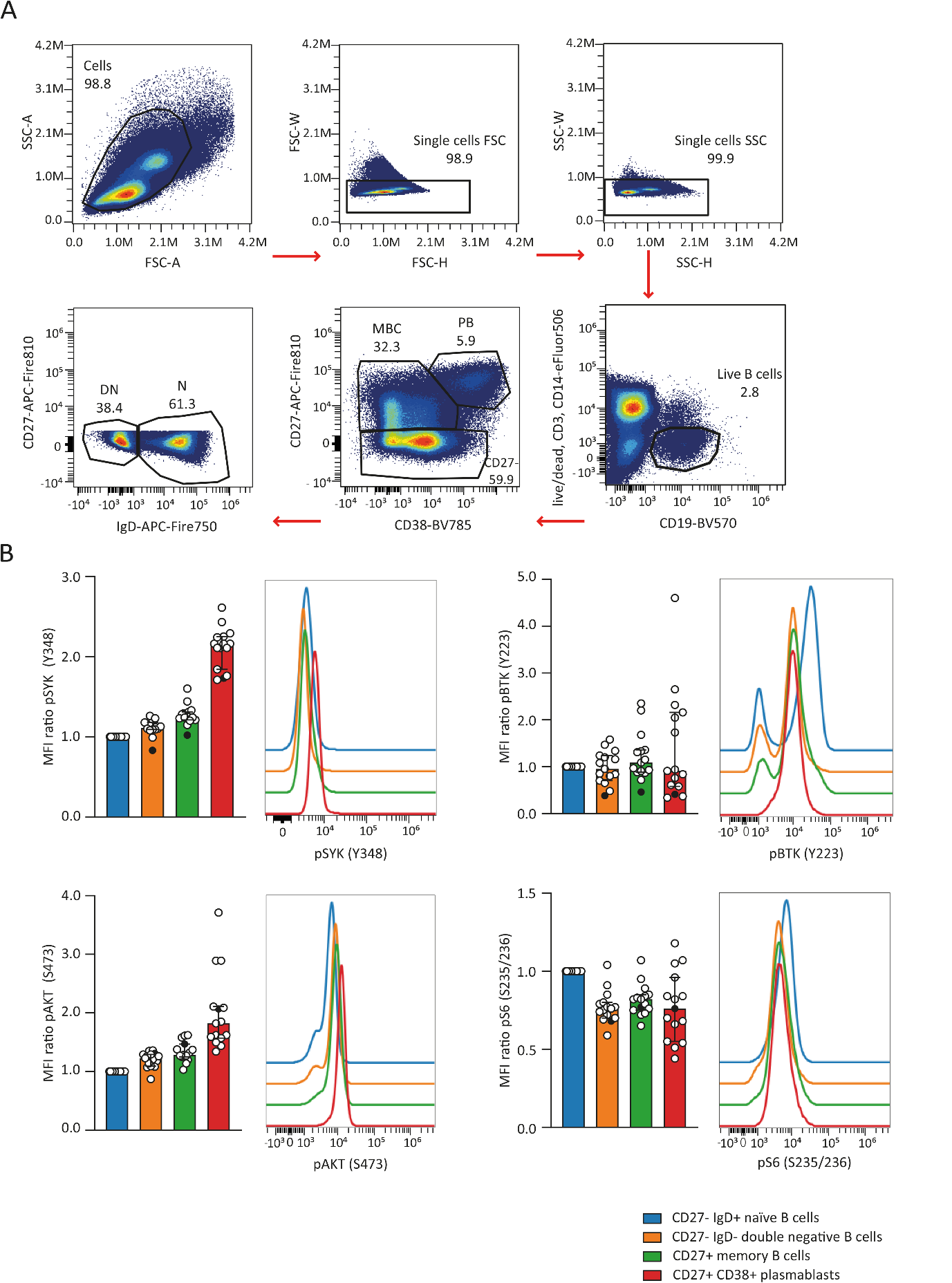


**Supplementary figure 1.** B-cell subset phosphorylation. **A.** Gating strategy for B-cell subset identification of one representative experiment. **B.** Bar graphs depicting phosphorylation levels, each dot represents one patient. Bar graphs show pooled data from 15 patients analyzed in separate experiments. Medians and interquartile ranges are indicated. Histograms depicting MFIs from one representative sample (black in bar graphs). pSYK and pS6 consisting of one peak, pBTK containing two peaks in all populations except PBs. pAKT showed a shoulder in the signal.


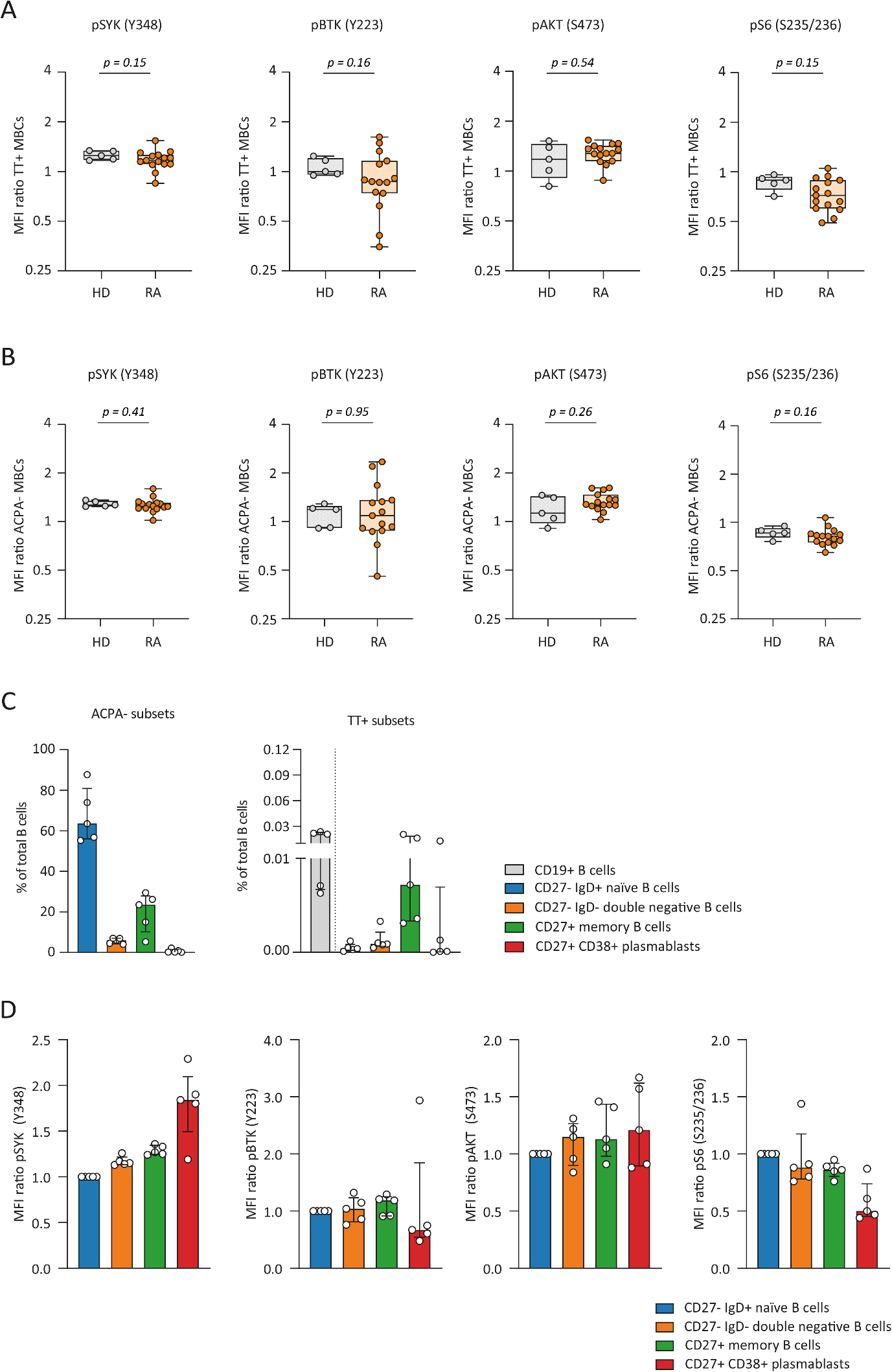


**Supplementary figure 2.** Phosphorylation of signaling molecules in B cells of healthy donors. **A.** Levels of pSYK, pBTK, pAKT and pS6 in TT-specific MBCs of healthy donors and RA patients. Whiskers depict min and max. **B.** Levels of pSYK, pBTK, pAKT and pS6 in ACPA-negative MBCs of healthy donors and RA patients. Whiskers depict min and max. **C.** Bar graphs depicting subset frequencies. Each dot represents one healthy donor. Figures show pooled data from five healthy donors analyzed in separate experiments. Medians and interquartile ranges are indicated. Dashed line emphasizes that CD19+ B cells (gray) are the total of the other populations. **D.** Bar graphs depicting phosphorylation levels, each dot represents one healthy donor. Bar graphs show pooled data from five patients analyzed in separate experiments. Medians and interquartile ranges are indicated. P-values were calculated with Mann-Whitney U test.

**
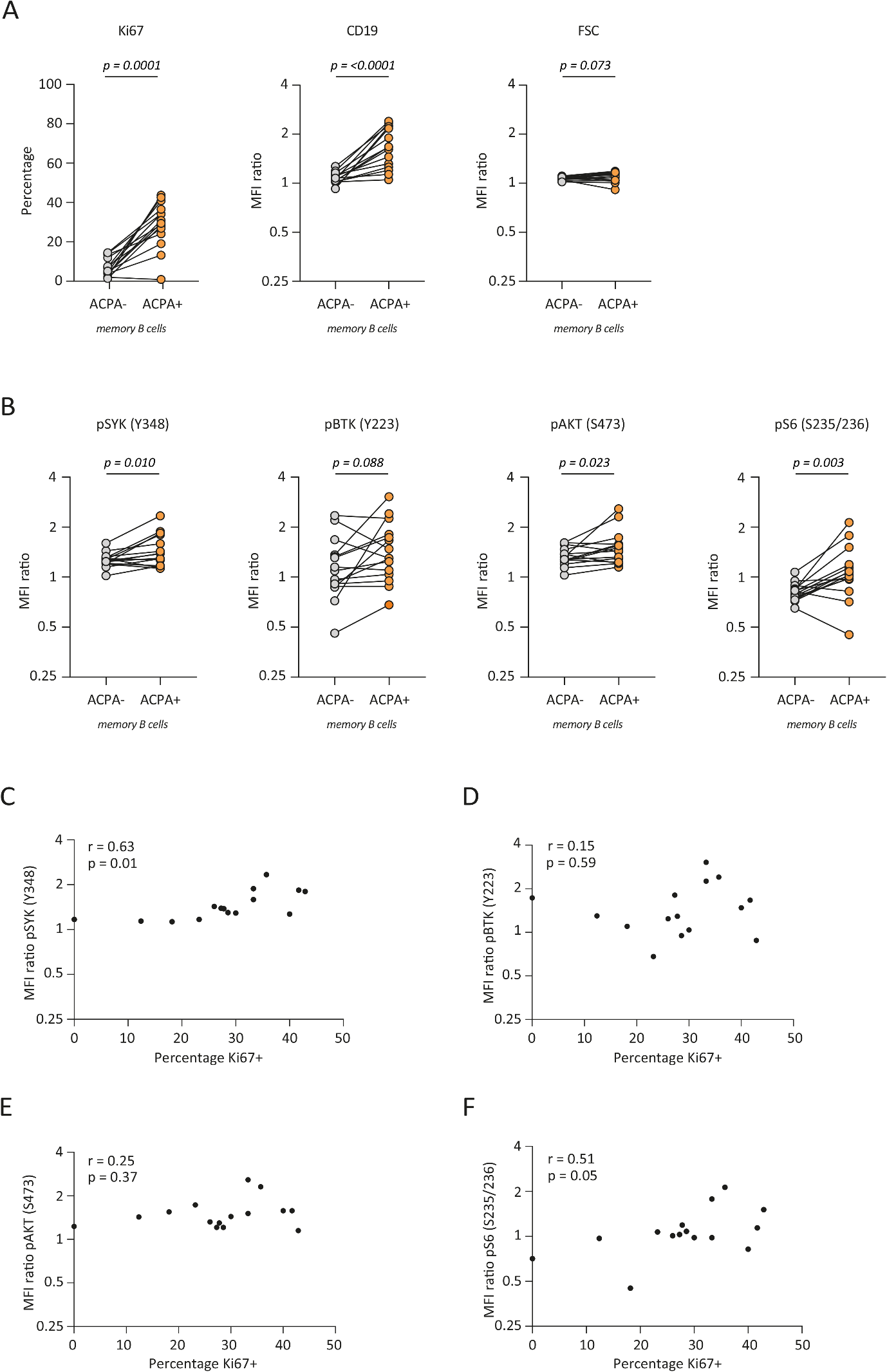
**

**Supplementary figure 3.** Phosphorylated signaling kinases in ACPA MBCs compared to ACPA-negative MBCs. **A.** Levels of Ki67 and MFI ratios of CD19 and FSC in ACPA MBCs. **B.** Levels of pSYK, pBTK, pAKT and pS6 in ACPA MBCs. **C-F.** Correlation plots for phosphorylated proteins and Ki67. Each dot represents one patient. P-values were calculated with Wilcoxon signed rank test (**A and B**). Pearson correlation analyses were performed (**C-F**).
